# Building Multisectoral Partnerships for Population Health and Health Equity

**Published:** 2010-10-15

**Authors:** Stephen Fawcett, Jerry Schultz, Jomella Watson-Thompson, Michael Fox, Roderick Bremby

**Affiliations:** University of Kansas, Lawrence, Kansas; Work Group for Community Health and Development; University of Kansas, Lawrence, Kansas; University of Kansas, Lawrence, Kansas; Kansas Department of Health and Environment, Topeka, Kansas

## Abstract

Poor performance in achieving population health goals is well-noted — approximately 10% of public health measures tracked are met. Less well-understood is how to create conditions that produce these goals. This article examines some of the factors that contribute to this poor performance, such as lack of shared responsibility for outcomes, lack of cooperation and collaboration, and limited understanding of what works. It also considers challenges to engaging stakeholders at multiple ecologic levels in building collaborative partnerships for population health. Grounded in the Institute of Medicine framework for collaborative public health action, it outlines 12 key processes for effecting change and improvement, such as analyzing information, establishing a vision and mission, using strategic and action plans, developing effective leadership, documenting progress and using feedback, and making outcomes matter. The article concludes with recommendations for strengthening collaborative partnerships for population health and health equity.

## The Problem

Poor performance in achieving population health goals is all too familiar. So is the accompanying every-decade ritual in the United States: the announcement of a new round of planning to create health goals for the nation (eg, Healthy People 2020), followed by a wave of enthusiasm and then disenchantment (eg, "the problems with the data arise from . . ."), search for the guilty (eg, "but they were never at the table"), punishment of the innocent (eg, "with this reorganization, our agency looks forward to . . ."), and reward for the uninvolved (eg, "we should never forget that America offers the world's highest-quality health care").

Lost in this drama are the numbers: for the 281 measurable public health performance objectives tracked for *Healthy People 2010*, only 10% met their targets (1). Although progress was made toward meeting nearly 50% (n = 138) of the objectives, 20% (n = 57) grew worse. Disparities in health outcomes for ethnic minorities also remain a failure. One of the most glaring disparities is in the African American community, in which 48% of adults suffer from a chronic disease compared with 39% of the general population. Why do we keep falling short of the bars we have set for ourselves in population health and health equity?

Several factors contribute to these poor results. First, multiple and unconnected sectors lack shared responsibility for outcomes. Consumers, providers, insurance companies, employers, and government agencies all vie for individual advantage in our fragmented health care system, avoiding responsibility for unimpressive outcomes. Second, the health care system lacks cooperation and collaboration in achieving population-level goals. Emmanuel and Fuchs (2) characterize this as "the myth of shared responsibility." Third, no public or private entity has overall responsibility for improving population health. This situation contributes to a willingness to proclaim victory for hard work, rather than meaningful improvement (3). Finally, moving toward improved population health and health equity requires understanding what works and what does not, and a willingness to agree on the price we pay for each. Sustained cooperation and shared responsibility among stakeholders in different sectors of a comprehensive public health system are necessary (4).

The public health response promotes community partnerships and cooperation as represented in the essential services. Public health agencies have come to recognize that community partnerships are a necessity in health improvement and that major health initiatives require community coalitions (5). Results are mixed, but the empirical evidence base for the effectiveness of partnerships to improve population health is growing (6-9).

In response to these problems, we offer a framework to guide collaborative action to improve population health. We also outline key processes for promoting community/system change and population health improvement. We conclude with 7 recommendations for strengthening collaborative partnerships for population health and health equity.

## Challenges in Building Collaborative Partnerships for Population Health

Collaboration is difficult to establish and maintain. First, stakeholders often have differing goals or understanding of the problem, which leads to disagreements and a devaluing of others' preferred strategies and approaches. Partners who share responsibility for naming and framing the problem may find it easier to bridge those differences. Having common goals makes it easier for stakeholders to see their potential contribution to healthier communities.

Second, stakeholders often focus narrowly on only a few of the many factors that contribute to the problem. They typically use interventions to address these through familiar channels of influence; yet improving population health requires comprehensive and coordinated approaches that address 1) multiple personal and environmental factors (eg, knowledge and skills, access to services and support, policies and living conditions), 2) multiple sectors (eg, health, education, government), 3) multiple ecologic levels (eg, individuals, organizations, communities, broader systems). Stakeholders are more likely to see the work they do as particularly needed; thus, shared responsibility among organizations working in multiple sectors is rare.

Third, working at multiple ecologic levels is challenging. Different determinants of health have different areas of policy action and related actors (eg, Medicare, federal officials; air quality, regional actors; school nutrition, local people). Few partnerships coordinate collaborative action across multiple levels. Fourth, working together requires flexibility on the part of stakeholders' organizations and those who fund them. Yet many nonprofit organizations and governmental agencies have policies that limit their capacity to share resources and responsibilities.

Fifth, measurement of accomplishments is also a challenge. Many initiatives do not have accurate or sensitive measures of success at the level of the whole community. Changes in the community or system — the unfolding of new programs and policies — need to be measured to see what was actually implemented and its contribution to more distant population-level outcomes. The merit of longer-term efforts is difficult to assess and adjust to without such measures of environmental change.

Sixth, incentives for population-level improvement, such as outcome dividends, are rare. Without effective incentives for improving population health, the time and effort of collaborating with partners may go unrewarded. Working together across organizations is challenging because of competition for limited funding. The prevailing contingencies of reinforcement help secure discrete resources for individual organizations, not groups of organizations to improve population-level outcomes for which responsibility is shared.

Seventh, our knowledge of how to effect change in communities and systems to produce substantial improvements in population health is limited. We need a better understanding of how key collaborative processes, such as action planning or community mobilization, can yield environmental changes that will improve population health. Stakeholders may lack the experience or training required to make the community or system changes needed to affect public health.

Finally, public health has promoted best practices or programs that work as a way to ensure that the most effective approaches are implemented. The problem is that evidence-based programs are typically tested with small numbers of individuals and evidence of comprehensive and context-appropriate strategies that actually improve population health is rare. Researchers and practitioners have begun to reorient their efforts to population health using frameworks and related processes (9-12).

## Framework and Processes for Collaborative Action

**Figure 1:**
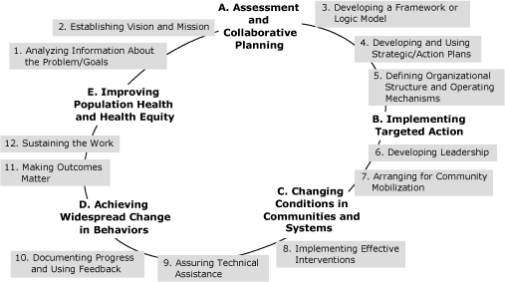
The sequential, iterative, and interactive components (A-E) of a framework that guides communities'  work to improve population health and 12 collaborative processes associated with the components. This framework is adapted from the Institute of Medicine framework for collaborative public health action (4).

We have adapted the Institute of Medicine framework for collaborative public health action in communities (Figure 1) (4,8). This framework, like other related frameworks (11), is iterative and interactive, with interdependencies between the phases and related processes. For instance, the first phase (assessment and collaborative planning) is oriented to indicators of success, such as reduced rates of childhood obesity or diabetes, that define the endpoints noted in the last phase (achieving improvement in population health and health equity). Emerging evidence suggests that 12 collaborative processes, such as action planning and making outcomes matter, may facilitate change and improve related outcomes in population health (Figure 1) (9,13,14).

### Assessment and collaborative planning

This first phase helps focus the attention of multisectoral collaborations on a common purpose. The process of *analyzing information* about candidate health concerns involves assessing strengths and problems (needs and resources) in the community (11,15). This process helps to pinpoint health concerns for priority attention and to identify those who may be able to contribute to the effort. This analysis often examines the related personal factors (eg, knowledge, skills, genetics) and environmental factors (eg, access, exposures, and opportunities; services and supports; policies) that influence population health outcomes. Critical analysis requires attention to social determinants of health, such as income inequality or social exclusion, that affect exposures and consequences and related disparities in population health outcomes. Through a multisectoral approach, representatives from different sectors of the community affected by the problem — such as health care providers, state or community organizations, business, and faith communities — are involved in naming the problem and goals related to the ultimate outcome. The process of *establishing a vision and mission* helps to communicate a common purpose that transcends the work of individual agencies and efforts (15,16).

*Developing a framework or logic model* helps clarify the approach used by the collaborative. It visually displays the expected pathway for how the effort will move from "here" (current level of the problem or goal) to "there" (changes in communities or systems and related improvements in priority population health outcomes) (15,17). The process of *developing and using strategic and action plans* further articulates how the community can move from vision and mission to attaining objectives (11,13,15). The planning process should include as agents of change those most affected by the issue, as well as those in a position to change communities and systems, such as leaders in business and government. Action planning should result in clearly identified changes to be sought in the community and system and who will do what by when to bring them about.

### Implementing targeted action

This second phase involves taking action to bring about community and system changes, including implementing different evidence-based programs and policies that may lead to population health improvement. The process of defining a clear *organizational structure and operating mechanism* is necessary to assure effective and sustainable multisectoral partnerships (16). Initiatives should identify explicit roles and responsibilities of partners, such as what community members and organizational leaders will do, to focus their actions on changing conditions that affect priority health outcomes.

*Developing effective leadership* for the multisectoral collaboration and its partners also is crucial since it enhances the capacity of an effort to mobilize for change and improvement (13,15). Leadership roles and responsibilities should be distributed across the partners to allow for ownership and responsibility for contributing to change and improvement in shared outcomes (18). *Arranging for community mobilization* involves designating people to support change efforts. This helps to assure accountability for changing programs and policies to be sought in different sectors (13,14).

### Changing conditions in communities and systems

The purpose of taking action is to facilitate changes in the community and broader system. Community/system changes refer to new or modified programs, policies, or practices facilitated by the collaborative partnership and related to its mission of improving population health. Changes in communities/systems are intermediate markers of success; discovering the conditions under which they are associated with improved outcomes in population health is a key research question for the field (19). *Implementing effective interventions, *those programs and strategies known to work, ensures that the partnership's comprehensive intervention can contribute to improvement in outcomes. *Assuring technical assistance* can increase the capacity of the multisectoral collaborations by enhancing core skills and knowledge to effectively implement key processes, such as action planning and community mobilization, and planned interventions such as evidence-based programs and policies (13). This phase should also address key social determinants of health such as income inequality and social exclusion that may contribute to disparities in health outcomes through differential exposures, vulnerabilities, and consequences.

### Changing behaviors and improving population health

The ultimate goal of multisectoral partnerships is to achieve widespread behavior change and improvement in population health outcomes and health equity. The process of *documenting progress and using feedback* allows for ongoing assessment of intermediate outcomes (community/system change) and population health outcomes to allow for adjustments (13,19). *Sustaining the work* through ongoing investment of activities and resources helps to ensure the continued viability of multisectoral collaborative partnerships.

Finally, the process of *making outcomes matter* involves using incentives to strengthen collaborative efforts (13,15). For instance, annual funding installments can be made contingent on evidence of progress; recognition and awards can be delivered for outstanding achievement; and tax incentives can be used to reward improvement in population health outcomes. The prevailing contingencies of reinforcement are typically too delayed, too small, and not contingent on performance. Group contingencies, such as outcome dividends or dollars returned to the community based on savings from improved outcomes, could be effective in sustaining collaborative action to improve population health. In a hypothesized community health and wellness system, the savings from improved population-level outcomes might be combined with other funding to help sustain the effective efforts of collaborative partnerships (20). In empirical case studies with community health coalitions, contingencies such as announcement of grant renewal contingent on evidence of changes in the community were associated with increased rates of documented changes (21). In a case study of outcomes-based contracting, contractors reported improved linking of funding investments and better accountability in a state health department's community partnership program (22).

Improvement in population health outcomes requires the continued engagement of 1) multiple agents of change (eg, community residents, state and local organizations), 2) working across sectors (eg, businesses, health care), 3) over time (eg, multiple years), and 4) across ecologic levels (eg, city, state). Multisectoral collaborations operate as complex adaptive systems that require interconnections to support effective and sustained efforts to change conditions. To promote change and improvement, differential consequences (ie, incentives and disincentives) also must take effect at levels corresponding to needed action (eg, community, state). Matching incentives with indicators of progress at appropriate levels could help maintain efforts of actors at different levels in changing communities and systems.

## Recommendations for Strengthening Population Health Partnerships

**Figure 2:**
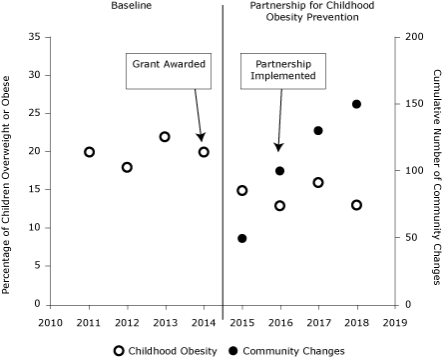
Hypothetical relationship between community changes (ie, every new or modified program, policy, or practice) facilitated by a partnership to prevent childhood obesity and associated improvement in a population-level outcome (ie, percentage of children who are obese or overweight). The vertical line indicates the start of the collaborative partnership to prevent childhood obesity.

We conclude with 7 key recommendations for strengthening collaborative partnerships to assure health for all:

1. *Establish monitoring systems to detect progress in achieving population health and health equity.* The public health infrastructure should ensure that data on indicators for all priority health concerns and related behavioral risk factors are made available to the public. Data should be available at regular intervals and at the level of those working together to promote health and health equity (eg, neighborhoods, rural communities). Monitoring systems should also report data for populations experiencing health disparities (eg, differences in outcomes associated with gender, race/ethnicity) and related social determinants of health.
2. *Develop and use action plans that assign responsibility for changing communities and systems.* Action plans should be developed that pinpoint specific changes in communities and systems to be sought — and who will do what by when to bring them about. Action plans change the ecology for engagement by highlighting opportunities for partners to bring about a new or expanded program or policy in those sectors in which they have the most influence.
3. *Facilitate natural reinforcement for people working together across sectors.* Principles of behavioral science suggest the importance of ensuring contingencies of reinforcement that are large and immediate enough for people to continue working together. For instance, arranging public recognition at group meetings, and media communications can help ensure that people's engagement in group efforts result in social and other forms of reinforcement.
4. *Assure adequate base funding for collaborative efforts that is sufficient to improve population-level outcomes.* Commitments of public and private foundation resources should be large and long enough to change conditions in communities and systems sufficiently to achieve the goal. For instance, to improve levels of physical activity enough to achieve outcomes of public health significance may require a base funding of $100,000 per year or more for at least 5 years.
5. *Provide training and technical support for those working in collaborative partnerships.* To ensure a competent workforce, training should be available in core competencies required for this work (23), including skills in assessment, planning, implementation, evaluation, advocacy, and developing partnerships across disciplines and sectors. This training should be widely available through interdisciplinary courses and Internet-based supports. For instance, the 7,000-page Community Tool Box (http://ctb.ku.edu) provides free access to training materials and just-in-time supports for collaborative action. Technical support should focus on implementation of key processes or mechanisms that affect the functioning of collaborative partnerships; for instance, in helping partnerships to develop and use action plans, document progress and use feedback, or make outcomes matter (13,14).
6. *Establish participatory evaluation systems for documenting and reviewing progress and making adjustments.* Participatory evaluation systems should be established to enable community and scientific partners to work together to monitor and reflect on what is happening. Data on community/system change help measure the intervention over time. Measurement of the amount and type of community/system change actually brought about (eg, by goal, duration, sector, change strategy, place) can help to estimate the potential effect of a collaborative partnership on outcomes (24). Online documentation systems can support review of rates of community/system change and associated contributions to population health improvement (19), as seen in the hypothetical relationship between community changes and associated improvement in a population health outcome (Figure 2). When online graphs of change efforts are accompanied by reflection, questions (eg, what are we seeing, what does it mean), and supports for improvement (eg, how to encourage participation or counter opposition), they can further support collaborative efforts (19).
7. *Arrange group contingencies to ensure accountability for progress and improvement.* Early in the collaborative partnership, group contingencies, such as annual renewal of grants for core support based on evidence of progress, should heighten group members' engagement in change efforts (24). In later years, group contingencies might take the form of bonus grants or outcome dividends for improvement in population health outcomes or reduced disparities (20). The size of the outcome dividend, the amount returned to the collaborative partnership, should reflect the estimated return on investment of demonstrated improvements in population health outcomes (eg, dollar savings from investments that reduce rates of obesity).

These recommendations aim to ensure conditions — including monitoring and feedback systems, training and technical support, and group incentives for progress — that can foster the success of broad collaborative partnerships (25). Such conditions should make it easier and more likely for multisectoral partnerships to achieve progress in improving population health and health equity.
